# Sex dimorphism in brain cell death after hypoxia-ischemia in newborn piglets

**DOI:** 10.1038/s41390-025-04046-5

**Published:** 2025-04-16

**Authors:** Daniel Alonso-Alconada, Marc Chillida, Ana Catalan, Pierre Gressens, Nicola J. Robertson

**Affiliations:** 1 Department of Cell Biology & Histology, School of Medicine & Nursing, University of the Basque Country (UPV/EHU), Leioa, Bizkaia Spain; 2 Psychiatry Department, OSI Bilbao-Basurto, Basurto University Hospital, Bilbao, Spain; 3 Neuroscience Department, University of the Basque Country (UPV/EHU), Leioa, Spain; 4 Biobizkaia Health Research Institute, Barakaldo, Spain; 5 CIBERSAM, Centro Investigación Biomédica en Red de Salud Mental, Madrid, Spain; 6 Université Paris Cité, NeuroDiderot, Inserm, Paris, France; 7 Institute for Women’s Health, University College London, London, UK; 8 Edinburgh Neuroscience & Centre for Clinical Brain Sciences (CCBS), University of Edinburgh, Edinburgh, UK

## Abstract

**Background:**

Clinical data suggest that females might be more resistant to hypoxia than males, with male sex recognized as a risk factor for suffering life-long neurological sequelae. However, the impact of hypoxia-ischemia in certain brain regions and its association with genetic sex remains unclear.

**Methods:**

Using the piglet model of neonatal brain injury, fifteen piglets (8 females and 7 males) were subjected to a global cerebral hypoxic-ischemic insult. After 48 h, total cell death and the number of necrotic, apoptotic and cleaved-caspase-3 positive cells was quantified in five brain regions.

**Results:**

Male piglets exposed to hypoxia-ischemia were more vulnerable than females (total cell death *p* < 0.01), also showing a region-specific response to brain injury depending on sex, with males being more affected in both deep gray (caudate *p* < 0.01; THAL *p* < 0.0001) and white (*p* < 0.01) matter. Despite necrosis was the primary form of cell death for both sexes, the pattern of cell death differed: while male piglets showed more necrosis (*p* < 0.0001), apoptosis (*p* < 0.0001) and caspase-3 activation (*p* < 0.0001) were higher in females.

**Conclusion:**

Our results suggest that male piglets were globally and regionally more vulnerable than females after HI; further, both the pattern of cell death and the apoptotic molecular mechanisms were sexually dimorphic.

**Impact:**

- Clinical data suggest that females might be more resistant to perinatal asphyxia than male newborns. The impact of hypoxia-ischemia in certain brain regions and the association of cell death patterns with sex remain unclear.
- Hypoxic-ischemic male piglets were more vulnerable than females, showing also increased regional vulnerability in both deep gray and white matter areas.
- Although necrosis was the primary form of cell death for both sexes, male piglets showed more necrosis, whereas apoptosis and caspase-3 activation were higher in females.
- Neonatal brain injury and therapeutic responses may be sex-dependent due to differences in cell death patterns and molecular mechanisms.

## Introduction

Current literature regarding sex differences in human brain morphology and neuronal physiology suggest that brain dimorphism starts in the fetal and newborn period and extends into adulthood.^1–4^

The influence of sex has been also described in neurological disorders^4^: whereas mental illnesses like schizophrenia or depression affect women preferentially, neurodevelopmental disorders show a higher prevalence in men. In neonates, epidemiological data suggest that females might be more resistant to hypoxia than males, as reported lower mortality rates from respiratory illnesses.^5,6^ In line with this, different prevalence between sexes also arises after perinatal asphyxia,^7^ with male sex recognized as a risk factor for suffering life-long neurological sequelae.^6^ However, a recent preliminary meta-analysis of human clinical trials reported that there is no significant sex effect in humans.^8^

Animal models are used to clarify the mechanisms underlying brain histological alterations and to search for new therapeutic approaches^9^; however, controversy exists when reviewing preclinical works evaluating the presence of sex dimorphism after hypoxic-ischemic insults. Some works suggest that male animals are more severely affected,^10,11^ other studies have reported no sex-differences in lesion size or tissue atrophy where post mortem neuropathology after hypoxia-ischemia was comparable across sex.^12–15^ Adding to the complexity, recent reports suggested that female rats exhibit greater histological damage.^16,17^

Similarly, the impact of hypoxia-ischemia in certain brain regions and its association with genetic sex remains unclear. Debate exists regarding the regional vulnerability due to sexual dimorphism, with some studies describing a reduction in cortical volume in males^18^ or increased injury in the striatum and white matter in female rodents.^17,19^ In neonates surviving intra-partum hypoxia-ischemia, the brain can be affected in a regionally specific manner depending on several factors, including the duration and severity of the hypoxia-ischemia: structural imaging has described typical patterns of basal ganglia and thalamic injury (Basal Ganglia and Thalamus (BGT)) with sentinel events and a watershed pattern of injury with chronic partial hypoxia-ischemia (for review, see refs. ^20,21^). A better knowledge about the possible effect of sex on vulnerability of brain regions to hypoxic-ischemic damage is needed.

Although hormones are a major contributor to sex-specific outcomes, they do not fully account for sex-specific responses to cerebral ischemia. The mechanisms of cell death and outcome of hypoxic-ischemic injury are influenced by sex.^22,23^ Divergent pathways are activated after a hypoxic-ischemic insult, completely independent of hormone exposure. For example, male cell death after stroke has been seen to activation of neuronal nitric oxide synthase with subsequent activation of PARP, whereas female cell death is triggered by cytochrome C and caspase activation.^13,24–26^

Here, we explored sex differences in neurologic damage after neonatal hypoxia-ischemia in newborn piglets; the piglet model provides a rich resource to assess safety and efficacy of neuroprotective therapies ready for clinical translation to the human newborn. There are key similarities in brain development and structure; the size of the piglet (~2 kg) allows the application of neurocritical care support with aEEG/EEG and full physiological monitoring with magnetic resonance spectroscopy (MRS) biomarkers as used in babies with neonatal encephalopathy. We evaluated whether male or female animals were more susceptible to hypoxic-ischemic-induced cell death, and whether there is a sexually dimorphic vulnerability to damage of certain brain regions.

## Materials and methods

All experimentation was in accordance with UK Home Office Guidelines (Animals [Scientific Procedures] Act 1986) and approved by the Animal Care and Use Committee of University College London Biological Services and Institute of Neurology.

### Animal experiments and surgical preparation

Fifteen piglets (8 females and 7 males) aged less than 30 h were anesthetized and surgically prepared. Following initial assessment for any signs of obvious infection including diarrhea and conjunctivitis, piglets were sedated with intramuscular midazolam (0.2 mg/kg), and arterial O_2_ saturation was monitored (Nonin Medical). Isoflurane anesthesia (4% vol/vol) was applied via a facemask during tracheostomy and intubation and was maintained (3% during surgery, 2% otherwise). Piglets were mechanically ventilated to maintain the arterial pressures of O_2_ (PaO_2_; 8-13 kPa) and CO_2_ (PaCO_2_; 4.5–6.5 kPa) allowing for temperature correction of the arterial blood sample.

An umbilical venous catheter was inserted to infuse maintenance fluids (10% dextrose, 60 ml/kg/d), fentanyl (3–6 μg/kg/h), and antibiotics (benzylpenicillin 50 mg/kg and gentamicin 2.5 mg/kg, every 12 h). An umbilical arterial catheter was inserted for continuous heart rate (HR) and mean arterial blood pressure (MABP) monitoring and 6-h blood sampling to measure PaO_2_, PaCO_2_, pH, electrolytes, glucose (3–10 mmol/l), and lactate (Abbott Laboratories). Bolus infusions of colloid (Gelofusin, B Braun Medical Ltd.) and inotropes maintained MABP > 40 mmHg. Arterial lines were maintained by infusing 0.9% saline solution (Baxter, 1 ml/h) with heparin sodium (1 IU/ml) to prevent line blockage. Both common carotid arteries were surgically isolated at the level of the fourth cervical vertebra and encircled by remotely controlled vascular occluders (OC2A, In Vivo Metric). After surgery, piglets were positioned prone in a plastic pod with their heads immobilized.

### Cerebral HI

A MRS surface coil was secured to the cranium, and the animal was positioned in a 9.4 Tesla Varian magnetic resonance spectrometer. While in the spectrometer, transient HI was induced by remote occlusion of both common carotid arteries, using inflatable vascular occluders, and reducing fraction of inspired oxygen (FiO_2_) to 12% (vol/vol).

During HI, cerebral energetics were monitored every 2 min by phosphorus (31P) MRS, and the β-nucleotide triphosphate (β-NTP; mainly ATP) peak height was automatically measured. When β-NTP peak height had fallen to 40% of baseline, FiO_2_ was adjusted to stabilize β-NTP at that level for 12.5 min. At the end of this 12.5-min period, the occluders were deflated and FiO_2_ was normalized. 31P spectra were acquired for a further 1 h to monitor recovery from HI. The time integral of the decrement of β-NTP/EPP (EPP = exchangeable phosphate pool = inorganic phosphate + phosphocreatine + (2γ + β)-NTP) during HI and the first 1 h of resuscitation quantified the acute energy depletion (AED). All animals received continuous physiological monitoring (SA instruments) and intensive life support throughout experimentation. Both groups were cared for over 48 h after HI and maintained normothermic (38–38.5 °C) throughout the entire experiment by using a warmed water mattress (Tecotherm) above and below the animal.

### Brain slices preparation

At 48 h, piglets were euthanized with pentobarbital, brains fixed by cardiac perfusion with cold 4% paraformaldehyde, dissected out and post-fixed at 4 °C in 2% paraformaldehyde for 7 days. 5mm-thick coronal slices of the right hemisphere, starting from anterior to the optic chiasma, were embedded in paraffin, sectioned to 5-µm thickness, and stained with H&E to validate the bregma for analysis.

**Fig. 1:**
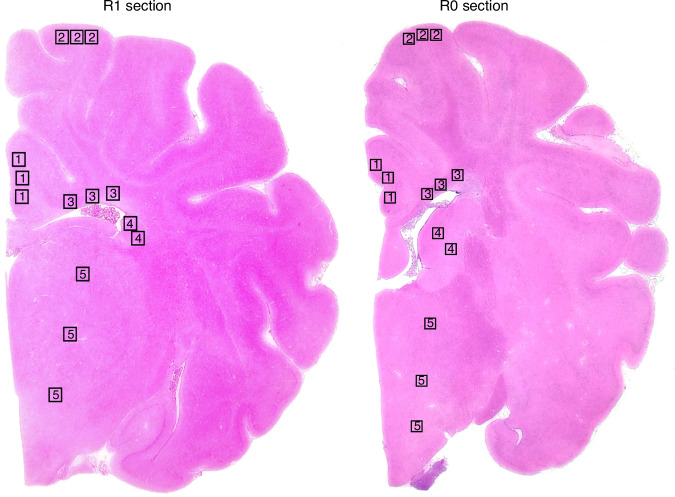
Histology of the piglet brain. Hematoxylin and Eosin-stained coronal sections of the piglet brain showing the regions assessed at bregma 0.0 (**R1 section**) and −2.0 (**R0 section**) brain levels sampled. 1. Cingulate Cortex; 2. Sensorimotor Cortex; 3. Periventricular white matter; 4. Caudate nucleus; 5. Thalamus.

For each animal, four sections at bregma 0 and −2.0 levels were evaluated. The regions of interest were five: the cingulate cortex (cTEX), the sensorimotor cortex (sTEX), the periventricular white matter (PvWM), the caudate nucleus (CDT) and the thalamus (THAL) (Fig. 1).

### Hematoxilin-Eosin (H&E) staining

5 µm brain sections embedded in paraffin were H&E-stained following the automated procedure by a Shandon Varistain® V24-4 (Thermo Electron Corporation) using Harris hematoxylin (Shandon Gill 2 Hematoxylin, Thermo Scientific) and eosin Y (Shandon Eosin-Y Alcoholic, Thermo Scientific). Finally, the samples were mounted with DPX histological mounting medium (#06522, Sigma-Aldrich) and left to dry for 24 h at room temperature.

### Immunohistochemistry

Paraffin-embedded sections were deparaffinised and rehydrated using the Shandon Varistain® V24-4 followed by antigen retrieval using a solution at pH 6 of 10 mM sodium citrate dihydrate (Sodium citrate dihydrate, W302600, Sigma-Aldrich), 0.05% TWEEN®20 (P1379, Sigma-Aldrich) and distilled water. Samples were boiled in this solution, kept for 20 min at 95–98 °C and allowed to cool at room temperature for 20 min. After several washes, endogenous peroxidase was blocked using a 1X solution of Phosphate Buffered Saline (PBS, Gibco®, Life Technologies) and 3% H_2_O_2_ (H/1800/15, Fischer Scientific) for 30 min. After blocking with 5% bovine serum albumin (Sigma-Aldrich), 0.4% Triton X-100 (Sigma-Aldrich) in 1X PBS for 1 h at room temperature, samples were incubated with the primary antibodies anti-cleaved caspase-3 (9661, Cell Signaling) diluted 1:100 in specific blocking buffer at 4 °C in the refrigerator overnight.

The next day, brain slices were incubated with the secondary antibody (1:500, goat anti-rabbit, biotin conjugate, 65-6140, Invitrogen) diluted in specific blocking buffer for 1 h at room temperature, followed by the ABC kit (1:500, Horseradish Peroxidase-Streptavidin Conjugate, 43-4323, Thermo Fisher Scientific) diluted in blocking buffer for 30 min at room temperature. After several washes, immunohistochemistry was revealed using a solution of diaminobenzidine (DAB Quanto, Epredia), for 5 min and controlled with the microscope. In the last step, sections were counterstained in a hematoxylin solution (Shandon Gill 2 Hematoxylin, Thermoscientific) for 5 s, dehydrated, and mounted using DPX.

### Image analysis

The histological images were obtained using an Olympus BX50F4 optical microscope. From each level (bregma 0.0 and −2.0), animal and section, 14 non-overlapping photographs were taken at X400 magnification of the five brain regions mentioned above: 3 photographs were taken for cTEX, sTEX, PVWM, and THAL, and 2 for CDT, as shown in Fig. 1.

Regional quantifications of total dead cells, necrotic, apoptotic, and cleaved caspase-3 positive cells were performed using Fiji/ImageJ 1.53e software by two blinded investigators. Cells with necrotic features were identified by a pyknotic nucleus or no nucleus, along with a swollen, eosinophilic cytoplasm, whereas apoptotic cells were characterized by the presence of nuclear karyorrhexis and low cytoplasmic change.^27^

### Statistical analysis

Samples were checked using the D’Agostino-Pearson normality test “omnibus K2” if followed a normal (Gaussian) distribution. To check the homogeneity of variances, the F test was used for *t*-test analyses, and the Brown-Forsythe test for ANOVA analyses. Parametric tests used were the *t*-test (for single comparisons) or ANOVA with a Tukey’s multiple comparisons test, and data presented as mean ± SD. In the case of failure to meet any of the requirements of normality or homogeneity of variances, Mann-Whitney (for single comparisons) and Kruskal-Wallis analyses with Dunn’s multiple comparisons test (replacing ANOVA) were used, and data presented as mean ± IQR. For data analysis, GraphPad Prism software version 8.0.1 was used. A *p* < 0.05 was considered significant.

## Results

### Insult severity

There were no intergroup differences in body weight (Females: 1.82 kg ± 0.22; Males: 1.77 kg ± 0.13) or in rectal temperature throughout the experimental protocol (Females: 38.5 °C ± 0.10; Males: 38.4 °C ± 0.12). The AED, the time integral of NTP/epp depletion relative to mean baseline during transient HI and the first hour of resuscitation, was measured to quantify acute insult severity. There was no significant difference (*p* = 0.39) in the hypoxic-ischemic insult severity between the two groups (AED-females: 0.09 ± 0.03; AED-males: 0.13 ± 0.04).

### Males showed higher counts of dead cells than females

**Fig. 2:**
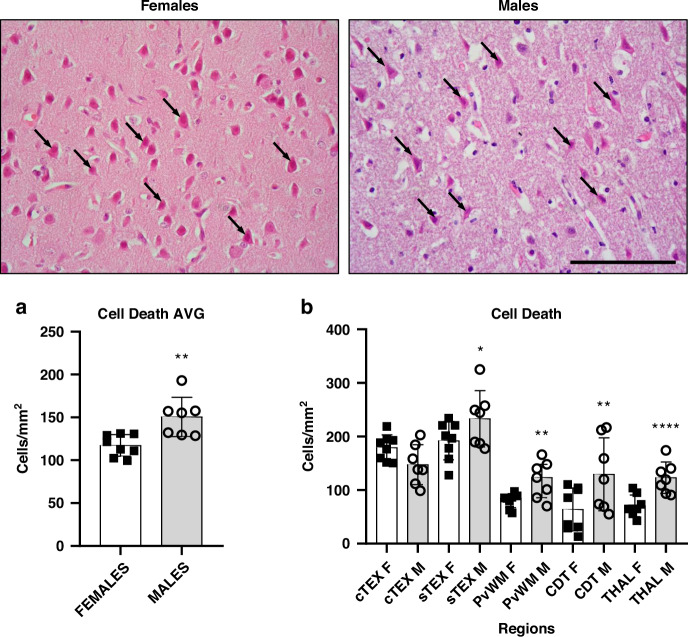
Representative H&E-stained microphotographs of the sensorimotor cortex from female and male piglets showing dead cells (arrows), with diffuse edematous matrix and hypodense neuropil (more relevant in males). Original magnification ×400. Scale bar: 100 μm. Graphs: **a** Averaged number of dead cells per mm^2^ (pooled across regions and bregma 0/−2.0 levels) from females and males. The overall number of dead cells per mm^2^ was significantly (Unpaired *t*-test, **p < 0.01) higher in males versus females. AVG average. **b** Quantitative results of regional cell death between females and males. cTEX cingulate cortex, sTEX sensorimotor cortex, PvWM periventricular white matter, CDT caudate nucleus, THAL thalamus. Unpaired *t*-test for all regions but PvWM, analyzed by Mann-Whitney. *p < 0.05 or **p < 0.01 or ****p < 0.0001 vs females.

The quantitative results of total cell death (global/overall and regional) and the comparison between females and males is shown in Fig. 2. The overall number of dead cells per mm^2^ (pooled across regions and bregma 0.0/-2.0 levels) was significantly (*p* < 0.01) higher in males versus females (Fig. 2a).

We observed significant higher cell counts of total dead cells (Fig. 2b) in the male sTEX (231.5 ± 120.6; *p* < 0.05), PvWM (119.4 ± 62.62; *p* < 0.01), caudate (127.3 ± 96.98; *p* < 0.01) and THAL (123.5 ± 61.74; *p* < 0.0001) compared to the same regions in females (sTEX: 192.9 ± 113.2; PvWM: 80.21 ± 65.08; caudate: 63.47 ± 57.05 and THAL: 69.81 ± 58.48 cells per mm^2^).

Tables 1 and 2 (Supplementary Tables) resume the interregional differences regarding total cell death, necrosis, apoptosis and cleaved caspase-3 counts for females (Supplementary Table 1) and males (Supplementary Table 2).

In females, regional assessment showed that the most affected regions were cTEX (178.2 ± 58.85) and sTEX (192.9 ± 113.2), whose counts doubled (*p* < 0.0001) those obtained for PvWM (80.21 ± 65.08), caudate (63.47 ± 57.05) or THAL (69.81 ± 58.48). No differences were found between PvWM, caudate, and THAL.

The male interregional analyses revealed that sTEX (231.5 ± 120.6) was the most damaged region after HI, with significant higher values of cell dead compared to cTEX (147.3 ± 102.3; *p* < 0.05), PvWM (119.4 ± 62.62; *p* < 0.001), caudate (127.3 ± 96.98; *p* < 0.01) or THAL (123.5 ± 61.74; *p* < 0.01). No differences were found between the other regions.

### Males have more necrosis than females

**Fig. 3:**
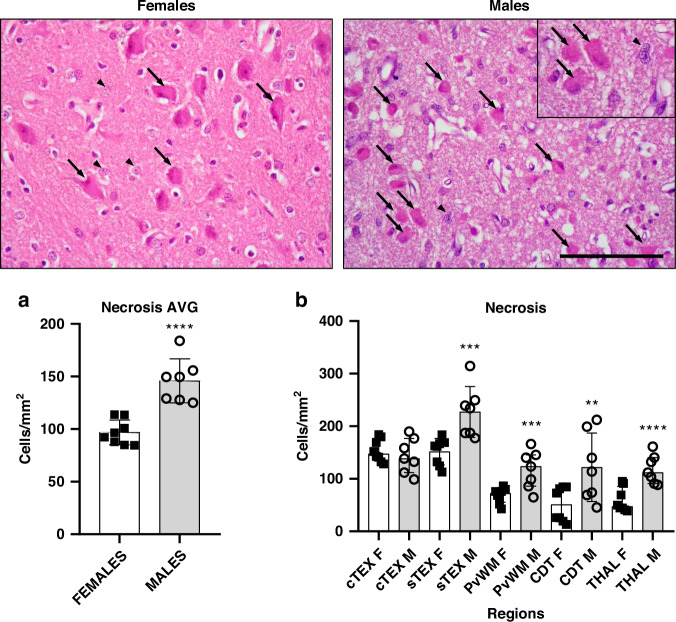
Representative H&E-stained microphotographs of the thalamus from female and male piglets showing necrotic-like (black arrows) and remaining living (arrowheads) cells. Original magnification: ×400. Inset magnification: ×630. Scale bar: 100 μm. Graphs: **a** Averaged number of necrotic cells per mm^2^ (pooled across regions and bregma 0/−2.0 levels) from females and males. The overall number of necrotic cells per mm^2^ was significantly (Unpaired *t*-test, ****p < 0.0001) higher in males versus females. AVG average. **b** Quantitative results of regional necrotic cell death between females and males. cTEX cingulate cortex, sTEX sensorimotor cortex, PvWM periventricular white matter, CDT caudate nucleus, THAL thalamus. Unpaired *t*-test for cTEX, sTEX, and THAL; Mann-Whitney for PvWM and CDT. **p < 0.01 or ***p < 0.001 or ****p < 0.0001 vs females.

Global necrosis assessment by H&E staining and regional quantification are shown in Fig. 3, comparing the results obtained for females and males. An increase in necrotic cell death (*p* < 0.0001) was observed for males as an overall effect of sex across all five areas examined (Fig. 3a). Regionally, necrotic cell values were significantly higher in males in 4 of the 5 regions: sTEX, PvWM, caudate and THAL (Fig. 3b).

The comparative study between regions of each sex (Supplementary Tables 1 and 2) revealed that in females, cTEX and sTEX were similarly affected by necrosis, with counts of ~150 cells per mm^2^. PvWM (67.99 ± 61.11), caudate (50.85 ± 56.65), and THAL (58.35 ± 56.50) showed significantly (*p* < 0.0001) lower values.

In males, the most damaged region by necrosis was sTEX (225.7 ± 116.0), whose counts were 1.5 times higher than those observed for cTEX (143.7 ± 100.4; *p* < 0.05), doubling the values of caudate (119.2 ± 92.85; *p* < 0.01), PvWM (118.0 ± 64.10; *p* < 0.001) and THAL (118.8 ± 59.32; *p* < 0.001). No differences were found between the other regions.

### Females have more apoptosis than males

**Fig. 4:**
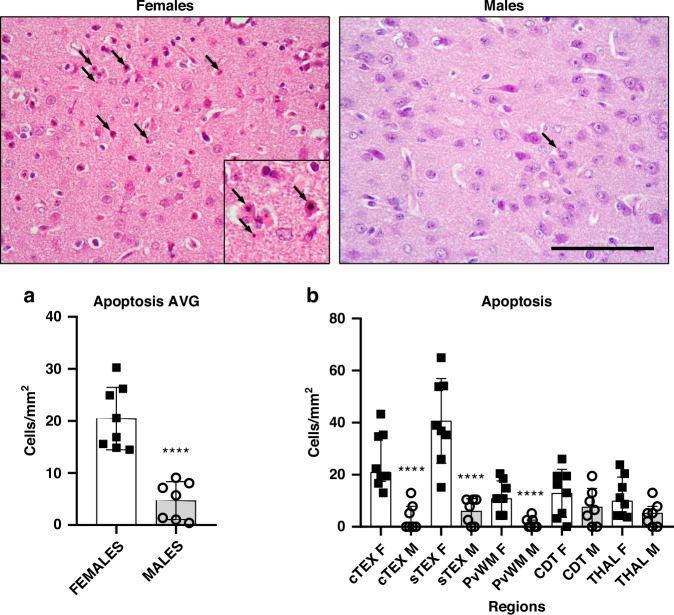
Representative H&E-stained microphotographs of the sensorimotor cortex from female and male piglets showing apoptotic-like (black arrows) cells. Original magnification: ×400. Inset magnification: ×630. Scale bar: 100 μm. Graphs: **a** Averaged number of apoptotic cells per mm^2^ (pooled across regions and bregma 0/−2.0 levels) from females and males. The overall number of apoptotic cells per mm^2^ was significantly (Unpaired *t*-test, ****p < 0.0001) lower in males versus females. AVG average. **b** Quantitative results of regional apoptotic cell death between females and males. cTEX cingulate cortex, sTEX sensorimotor cortex, PvWM periventricular white matter, CDT caudate nucleus, THAL thalamus. Unpaired *t*-test for cTEX, CDT, and THAL; Mann-Whitney for sTEX and PvWM. ****p < 0.0001 vs females.

The quantitative results of apoptotic cell death appear detailed in Fig. 4. The average value across the five regions included in this work showed that apoptosis in females is more prominent (*p* < 0.0001) than in males (Fig. 4a).

In the interregional study between females and males, we observed significant (*p* < 0.0001) higher counts of apoptotic cells in the female cTEX, sTEX, and PvWM brain regions compared to the same areas in males (Fig. 4b).

In the comparison between regions (Supplementary Tables 1 and 2), the female sTEX (41.27 ± 53.63) was the most affected area by apoptosis, with higher values than PvWM (12.22 ± 16.31; *p* < 0.001), caudate (12.62 ± 16.16; *p* < 0.05) and THAL (11.46 ± 18.12; *p* < 0.001). The other cortical region, the cTEX (25.48 ± 32.88), also showed more apoptotic cell death (*p* < 0.05) than PvWM and THAL in female piglets.

In male piglets, no differences in regional apoptosis were found, neither in cortical areas nor in deep gray or white matter regions.

### Females have more cleaved caspase-3 positive cells than males

**Fig. 5:**
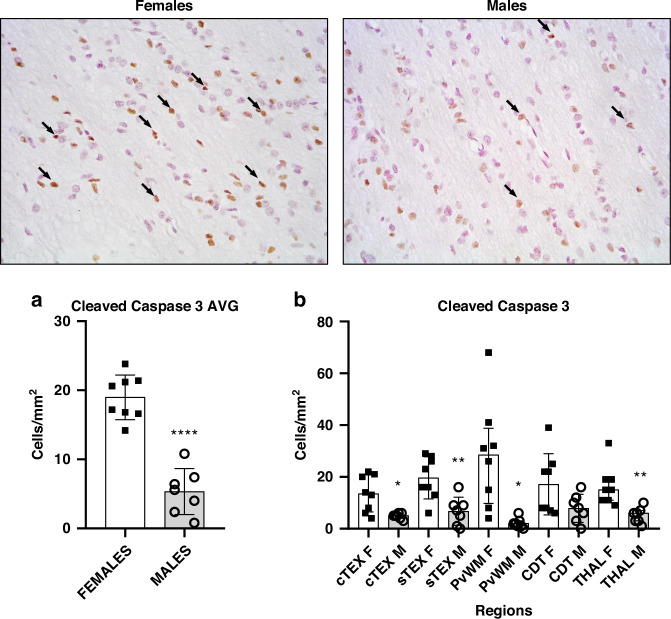
Representative microphotographs of cleaved caspase-3 immunostaining and hematoxylin counterstain of the periventricular white matter from female and male piglets showing cleaved caspase-3 positive cells (black arrows). Original magnification: ×400. Scale bar: 100 μm. Graphs: **a** Averaged number of cleaved caspase-3 positive cells per mm^2^ (pooled across regions and bregma 0/−2.0 levels) from females and males. The overall number of cleaved caspase-3 positive cells per mm^2^ was significantly (Unpaired *t*-test, ****p < 0.0001) lower in males versus females. AVG average. **b** Quantitative results of regional evaluation of cleaved caspase-3 positive cells between females and males. cTEX cingulate cortex, sTEX sensorimotor cortex, PvWM periventricular white matter, CDT caudate nucleus, THAL thalamus. Unpaired *t*-test for sTEX and CDT; Mann-Whitney for cTEX, PvWM and THAL. *p < 0.05 or **p < 0.01 vs females.

The quantitative results for cleaved caspase-3 are shown in Fig. 5. The overall number of cleaved caspase-3 positive cells per mm^2^ (pooled across regions and bregma 0/-2.0 levels) was significantly (*p* < 0.0001) higher in females (Fig. 5a).

When comparing by sex, cleaved caspase 3 positive counts were significantly higher in females in 4 of the 5 regions. We observed significant higher cell counts of caspase-3 positive cells (Fig. 5b) in the female cTEX (13.44 ± 7.03; *p* < 0.05), sTEX (19.56 ± 8.17; *p* < 0.01), PvWM (28.11 ± 20.44; *p* < 0.01) and THAL (16.56 ± 7.66; *p* < 0.0001) compared to the same regions in males (cTEX: 4.83 ± 1.16; sTEX: 6.66 ± 5.95; PvWM: 2.16 ± 2.13; THAL: 5.16 ± 3.06 cells per mm^2^).

Regional assessment showed no significant differences between any region in females (Supplementary Table 1). Similarly, we found no statistical differences in regional cleaved caspase 3 counts in males (Supplementary Table 2).

## Discussion

Using the piglet model of neonatal brain injury, we evaluated whether females or males were more susceptible to hypoxia-ischemia-induced cell death and explored possible sexually dimorphic vulnerability to damage of certain brain regions. We also determined, in a sex-dependent manner, the global and regional response in the pattern of cell death. In brief, (i) male piglets exposed to hypoxia-ischemia and subsequently maintained in normothermia were more vulnerable than females, (ii) there was a region-specific response to brain injury depending on sex, with males being more affected in both deep gray and white matter areas and (iii) despite necrosis was the primary form of cell death for both sexes, the pattern of cell death differed between sexes: while males showed more necrosis, apoptosis and caspase-3 activation were more prominent in female piglets.

The piglet model has strong similarities to newborn infants with neonatal encephalopathy regarding the timing of the evolution of injury, neuropathology, and pattern of injury after hypoxia-ischemia.^28,29^ As sex dimorphism after neonatal hypoxia-ischemia was not yet examined in a large animal model (all previously commented works were carried out using rats or mice), we used the newborn piglet to explore this. Piglets did not receive any treatment, being maintained in normothermic conditions (38.5 °C) throughout the experiment, so these histological results are a direct representation of the neurotoxic cascade after the hypoxic-ischemic insult. We observed that male piglets were more vulnerable to hypoxia-ischemia, showing a global 50% increase in total cell death when comparing to females. These results are in accordance with clinical data demonstrating a higher vulnerability in male infants towards hypoxic-ischemic brain injury, which in turn will increase the incidence of long-term neurological deficits compared to females with similar brain injury.^6,7^

The pattern of brain injury in NE depends on multiple factors such as brain maturation, severity of hypoxia and/or ischemia, cerebral blood flow, health and nutritional status of the fetus, inflammatory status and duration of the insult.^30^ The early primate neonatal models showed that the pattern of brain injury after hypoxia-ischemia is affected by the severity and duration of HI. Two distinct patterns of injury were described in the 1950s-1970s – acute total asphyxia^31^ and chronic partial asphyxia.^32^ The typical patterns of brain injury as seen on conventional brain MRI in neonates are reviewed by Wisnowski et al.^20^ and Parmentier et al.^21^; the BGT pattern is associated with acute total asphyxia (typically sentinel events) and the watershed predominant pattern associated with chronic partial asphyxia. The near total global pattern is the most severe, characterized by diffuse injury.

In this study we explored if certain brain regions are more vulnerable to HI depending on sex. The brain anatomy of the piglet resembles more closely the human brain than rats/mice, with similar gray and white matter patterns in human and piglet brains.^33,34^ Further, the brain growth spurt occurs around birth in the piglet, similar to the human and unlike the precocial and postnatal brain growth spurt seen in other models.^35^ In this model, we have previously reported extensive neuronal death in cortical, deep gray, and PvWM of hypoxic-ischemia animals.^36^ Here, we analyzed five brain regions encompassing cortical gray matter (cTEX and sensorimotor cortex), deep gray matter (CDT and THAL), and PvWM. As expected, all five brain regions showed relevant cell death after the insult for both sexes.

Noteworthy, the male brain displayed higher counts of dead cells in four of the five regions studied when compared to the female brain: sensorimotor cortex, CDT, THAL, and PvWM. Our regional results are in line with previous reports where murine males were more severely affected in the cortex, striatum, and white matter,^10,11,25^ a regional vulnerability followed by worse neurological outcomes.^37^ Here, deep gray and white matter areas were differently affected depending on sex, with the male piglet brain showing a worse response in both patterns of brain injury. These results may correlate with the neurological outcome described in human newborns, where cerebral palsy and related disorders during perinatal development are more common in male newborns^6^, and a cognitive advantage in females has been described after the hypoxic-ischemic brain injury.^14^

As the morphology of the degeneration seen in the piglet forebrain mirrors more faithfully the pattern of the human newborn than the patterns of neurodegeneration seen in rodents,^34^ we next wondered if the pattern of cell death could also be sexually dimorphic in the newborn piglet.

Characterized by cell swelling and membrane rupture, necrosis has been documented as the major cell death phenotype in the brain following neonatal hypoxic-ischemic injury in human infants^38^ and piglets,^33^ so we first analyzed the global presence and made regional quantifications of this cell death phenotype. Necrotic cell death was highly prevalent in both sexes, with average values ranging from ~100 cells/mm^2^ in females to 150 cells/mm^2^ for males. Again, we found a selective vulnerability to total necrotic-cell death in male piglets, of particular relevance in brain regions like sensorimotor cortex, CDT, THAL and PvWM.

There are few studies evaluating sex differences in necrosis, as the majority of evidence has been focused on apoptotic pathways. Necrosis, mitochondrial collapse, and oxidative stress are tightly linked after hypoxia-ischemia,^34^ and sex may be a variable to take into consideration: mitochondrial respiration has shown to be two-fold more impaired in male rats, which added to a lower antioxidant capacity, leading to regional increases in oxidative stress in injured brain males, but not females.^39^ Therapies targeting oxidative stress to reduce brain damage after hypoxia-ischemia have revealed different responses to medications in males and females. After allopurinol^40^ or N-acetylcysteine^41^ administration, the reduction in brain damage was sex-dependent, with females showing decreased brain infarct as compared to males; however, inhalation of nitric oxide protected males but not females from neonatal hypoxia-ischemia in the mouse brain.^25,26^ These results highlight that further research is needed considering sex in neurotherapeutics to achieve wider levels of protection against necrosis/brain damage.

Considered as a type of delayed cell death, apoptosis often occurs hours-to-days following the initial hypoxic-ischemic insult, thus neuroprotective strategies mostly try to target it during the therapeutical window. Here, we also observed a sex-dependent effect on apoptosis in the piglet brain, despite necrosis was still the primary form of cell death. Apoptotic profiles were greater in female brains than in male brains, the latter showing very low counts of apoptotic-like cells in most regions. As described before,^39^ females’ capacity to regulate mitochondrial impairment and oxidative stress may lead to a “more orderly” pattern of cell death, instead of the predominance of necrosis showed in males. As a limitation of this work, we acknowledge that apoptosis may continue beyond 48 h as in rodent models.^42^ However, in our study, logistical constraints related to the intensive care required for piglets prevented extending the survival period beyond 48 h. Additionally, piglets have a different developmental trajectory compared to rodents, and apoptosis progression may not be directly comparable. Future studies to examine longer recovery times in piglets are needed, potentially incorporating additional time points to track the evolution of cell death processes over time. The biochemistry of apoptotic pathways has been extensively discussed, and a sex effect suggested.^43^ Data in rodents have described that females appear to more specifically rely on the caspase-dependent apoptotic pathway (as showed here), whereas male cells appear to die, however, via the caspase-independent pathway. The activation of different molecular apoptotic pathways may be fundamental to sex differences in brain injury and repair and, as a consequence, sex might influence the outcome of neuroprotective intervention strategies, with a number of therapies being only effective to one sex or the other.

This work has some limitations. As previously demonstrated in rodents, isoflurane anesthesia can enhance apoptosis in the immature brain. In our study, both male and female groups were subjected to the same anesthetic regimen, minimizing potential confounding effects within the dataset. However, as we did not include a control group of piglets exposed solely to anesthesia without HI, we cannot completely rule out an influence of anesthesia on apoptotic cell counts based on sex. Although the number of animals used in large animal studies cannot match those from rodent studies, the sample size of this work may be a study limitation; however, we found the effect size to be considerable demonstrating significant differences between the groups, in agreement with previous works from our group.^44,45^ The severity of the hypoxic-ischemic insult measured by the ^31^P MRS AED was similar between male and females, however we could not control for regional blood flow and the heterogeneity of the intrinsic response to injury.

## Conclusions

Our results suggest that male piglets were globally and regionally more vulnerable than females after HI; both the pattern of cell death and the apoptotic molecular mechanisms were sexually dimorphic.

Future research will need to assess the influence of sex on neonatal brain injury and response to therapies (as one sex might be more protected than the other), so that individualized and more precise neuroprotective therapies can be developed.

## Supplementary information

Supplementary Material

## Data Availability

All data included and/or analyzed in this study are available upon reasonable request from the corresponding author.
