# Supplementary material for: Sex dimorphism in brain cell death after hypoxia-ischemia in newborn piglets
Source: Pediatr Res. 2025 Apr 16;98(3):1120–7. doi: 10.1038/s41390-025-04046-5 (PMC12507683; doi:10.1038/s41390-025-04046-5)
Supplement: Supplementary file 1 — Supplementary Material [file 41390_2025_4046_MOESM1_ESM.pdf]

Table 1. Comparison between regions for total cell death, necrosis, apoptosis and cleaved caspase 3 positive counts cells in female piglets. MRD: Mean Rank Difference.

	**Cell death**		**Necrosis**		**Apoptosis**		**Cleaved Caspase-3**	
	MRD	P value	MRD	P value	MRD	P value	MRD	P value
**cTEX vs. sTEX**	3.28	>0.9999	18.36	>0.9999	-18.29	>0.9999	-6.11	0.8187
**cTEX vs. PvWM**	86.21	<0.0001	84.59	<0.0001	38.89	0.0288	-14.67	0.0940
**cTEX vs. CDT**	97.16	<0.0001	100.40	<0.0001	28.07	0.5442	-3.67	0.9667
**cTEX vs. THAL**	93.35	<0.0001	92.15	<0.0001	37.19	0.0438	-3.11	0.9817
**sTEX vs. PvWM**	82.93	<0.0001	66.23	<0.0001	57.19	0.0001	-8.56	0.5663
**sTEX vs. CDT**	93.89	<0.0001	82.02	<0.0001	46.36	0.0149	2.44	0.9926
**sTEX vs. THAL**	90.08	<0.0001	73.78	<0.0001	55.48	0.0002	3.00	0.9840
**PvWM vs. CDT**	10.96	>0.9999	15.79	>0.9999	-10.82	>0.9999	11.00	0.3171
**PvWM vs. THAL**	7.15	>0.9999	7.559	>0.9999	-1.71	>0.9999	11.56	0.2704
**CDT vs. THAL**	-3.81	>0.9999	-8.24	>0.9999	9.12	>0.9999	0.56	>0.9999

Table 2. Comparison between regions for total cell death, necrosis, apoptosis and cleaved caspase 3 positive counts cells in male piglets. MRD: Mean Rank Difference.

	**Cell death**		**Necrosis**		**Apoptosis**		**Cleaved Caspase-3**	
	MRD	P value	MRD	P value	MRD	P value	MRD	P value
**cTEX vs. sTEX**	-34.92	0.0231	-35.32	0.0206	-11.47	>0.9999	-1.83	0.9382
**cTEX vs. PvWM**	12.68	>0.9999	11.64	>0.9999	4.89	>0.9999	2.67	0.7983
**cTEX vs. CDT**	9.30	>0.9999	11.15	>0.9999	-19.80	0.3288	-3.00	0.7218
**cTEX vs. THAL**	9.36	>0.9999	10.35	>0.9999	-8.74	>0.9999	-0.33	>0.9999
**sTEX vs. PvWM**	47.60	0.0003	46.96	0.0004	16.36	0.4870	4.50	0.3545
**sTEX vs. CDT**	44.22	0.0056	46.47	0.0029	-8.33	>0.9999	-1.17	0.9879
**sTEX vs. THAL**	44.28	0.0011	45.67	0.0007	2.74	>0.9999	1.50	0.9695
**PvWM vs. CDT**	-3.38	>0.9999	-0.49	>0.9999	-24.69	0.0781	-5.67	0.1584
**PvWM vs. THAL**	-3.32	>0.9999	-1.29	>0.9999	-13.63	>0.9999	-3.00	0.7218
**CDT vs. THAL**	0.06	>0.9999	-0.81	>0.9999	11.06	>0.9999	2.67	0.7983
